# Methadone-Induced Delayed Posthypoxic Encephalopathy: Clinical, Radiological, and Pathological Findings

**DOI:** 10.1155/2010/716494

**Published:** 2010-12-09

**Authors:** Manoj Mittal, Yunxia Wang, Alan Reeves, Kathy Newell

**Affiliations:** ^1^Department of Neurology, The University of Kansas Medical Center, Kansas City, KS 66160, USA; ^2^Department of Radiology, The University of Kansas Medical Center, Kansas City, KS 66160, USA; ^3^Department of Pathology, The University of Kansas Medical Center, Kansas City, KS 66160, USA

## Abstract

*Objective*. To describe the clinical, radiological and pathological findings in a patient with methadone-induced delayed posthypoxic encephalopathy (DPHE). *Case Report*. A Thirty-eight-year-old man was found unconscious for an unknown duration after methadone and diazepam ingestion. His initial vitals were temperature 104 degree Fahrenheit, heart rate 148/minute, respiratory rate 50/minute, and blood pressure 107/72 mmhg. He developed renal failure, rhabdomyolysis, and elevated liver enzymes which resolved completely in 6 days. After 2 weeks from discharge he had progressive deterioration of his cognitive, behavioral and neurological function. Brain MRI showed diffuse abnormal T2 signal in the corona radiata, centrum semiovale, and subcortical white matter throughout all lobes. Extensive work up was negative for any metabolic, infectious or autoimmune disorder. Brain biopsy showed significant axonal injury in the white matter. He was treated successfully with combination of steroids and antioxidants. Follow up at 2 year showed no residual deficits. *Conclusion*. Our observation suggests that patients on methadone therapy should be monitored for any neurological or psychiatric symptoms, and in suspected cases MRI brain may help to make the diagnosis of DPHE. A trial of steroids and antioxidants may be considered in these patients.

## 1. Introduction

Delayed posthypoxic encephalopathy (DPHE) is a rare clinical entity characterized by delayed neurological deficits seen after an initial hypoxic-ischemic insult. It is commonly described in relation to carbon monoxide (CO) poisoning with prevalence of 0.06%–2.8% and equal sex predilection [1]. Prognosis can be variable, ranging from complete recovery to death. Methadone-induced DPHE is very rare and has been reported in only 5 cases so far: 4 children and 1 adult [2–6]. We report clinical, radiological, and pathological findings of methadone induced DPHE in a 38-year-old man who was successfully treated with methylprednisolone.

## 2. Case Report

A 38-year-old righthanded Caucasian male computer engineer presented to the Kansas University Medical Center emergency department (ED) after being unconscious for an unknown duration. His history was notable for prior back and neck pain, for which he had taken methadone and diazepam, prescribed for his father. He had a long history of alcohol abuse, smoking, and hypertension. He had no drug allergies. Family history was notable for a father with schizophrenia, depression, and stimulant abuse. He was unresponsive and comatose on initial examination. His pupils were small (2 mm) and reactive. All four extremities moved in response to painful stimuli, and plantar responses were bilaterally downgoing. He had right basal crackles. His initial vital signs showed a temperature of 104 degree Fahrenheit, heart rate of 148 beats/minute, a respiratory rate of 50/minute, and blood pressure of 107/72 mmhg. Patient did not undergo any cardiopulmonary resuscitation and was intubated for airway protection. Postintubation ABG showed pH 7.4 (reference range (RR): 7.35–7.45), pCO_2_ 37 (RR: 35–45 mmhg), pO_2_ 110 (RR: 85–100 mmhg), and bicarbonate 23 (RR: 22–26 MEQ/L). His blood pressure dropped (78/50 mmhg) requiring intravenous dopamine. Initial blood tests revealed a white blood cell count of 13.1 (RR: 4.5–11.0 K/UL), aspartate transaminase of 233 (RR: 7–40 IU/L), alanine transaminase of 88 (RR: 7–56 IU/L), troponin of 3.98 (RR: 0.0–0.05 NG/ML), and remarkably elevated CPK 3339 (RR: 22 to 198 IU/L). The patient was in acute renal failure with BUN 13 (RR: 8–20 MG/DL), creatinine of 3.2 (RR: 0.4–1.24 MG/DL), and potassium of 6.6 (RR: 3.5–5.1 MMOL/L). Urine toxicological screen was positive for methadone and benzodiazepines, and negative for amphetamines, barbiturates, cannabinoids, cocaine and phencyclidine. His initial CSF exam showed protein 37.2 (15–40 MG/DL), glucose 81 (RR: 40–75 MG/DL), white cells 3 (RR: 0–5/UL), red cells 1 (RR: <1/UL), myelin basic protein (MBP) 2.8 (RR: <1.5 NG/ML), negative cultures, and no oligoclonal bands. A CAT scan without contrast of the head showed loss of distinction between grey and white matter. Patient did not have an initial MRI. Coronary angiogram revealed no abnormalities. 

Despite no growth on cultures, he was empirically treated for possible aspiration pneumonia with intravenous antibiotics. He received kayexalate and intravascular hydration along with sodium bicarbonate for acute renal failure. His condition stabilized, and he was extubated after 2 days. He gradually recovered over the next 4 days and was subsequently discharged home after 6 days of hospitalization. At discharge, he had no neurological deficits, and he was able to return to his previous profession as a computer engineer.

However, 2 weeks after discharge, his cognitive and behavioral function began to deteriorate. He was brought to the ED 34 days from initial presentation, for subacute onset of difficulties with short-term memory, confusion, impairment of executive function, and lack of motivation. He was admitted to the psychiatry ward. At the time of the second admission, he was alert and oriented to person only. He was able to follow one-step commands. Cranial nerves were intact. He was moving all his extremities spontaneously. He had positive grasp and palmomental reflexes and was hyperreflexic with bilateral plantar extensor response. He continued to deteriorate over the next few days to the point he was totally dependent on others for all activities of daily living.

CSF cytology, protein, cell counts, and glucose were normal. Oligoclonal bands were not detected. MBP was 4.4 (RR: <1.5 NG/ML). CSF 14-3-3 protein was negative. Urine sulfatide, peripheral leukocyte arylsulfatase A assay, serum long chain fatty acids, ELISA for HIV, serum levels of vitamin B12, methylmalonic acid, folate, TSH, vitamin E level, and thiamine were all within normal range. First EEG was done 46 days from initial presentation and showed symmetric bihemispheric dysfunction, and second EEG done on day 60 showed well-developed dominant rhythm of 10  hertz present mainly in the left hemisphere, marked depression of alpha, and medium amplitude of 2-3  hertz waves in the right frontotemporal regions, suggesting possible dysfunction of the right hemisphere. There was no indication of epileptic discharges.

**Figure 1:**
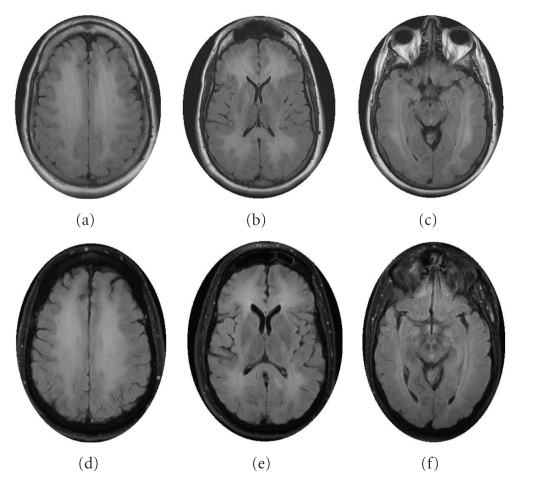
Initial brain MRI illustrates diffuse abnormal T2 FLAIR signal in the corona radiata, centrum semiovale, and subcortical white matter throughout all lobes (a)–(c). Followup MRI brain at 2 months was unchanged (d)–(f).

MRI of the brain revealed diffuse abnormal T2 signal in the corona radiata, centrum semiovale, and subcortical white matter throughout all lobes. There was no abnormal contrast enhancement, and no foci of restricted diffusion (Figure 1).

**Figure 2:**
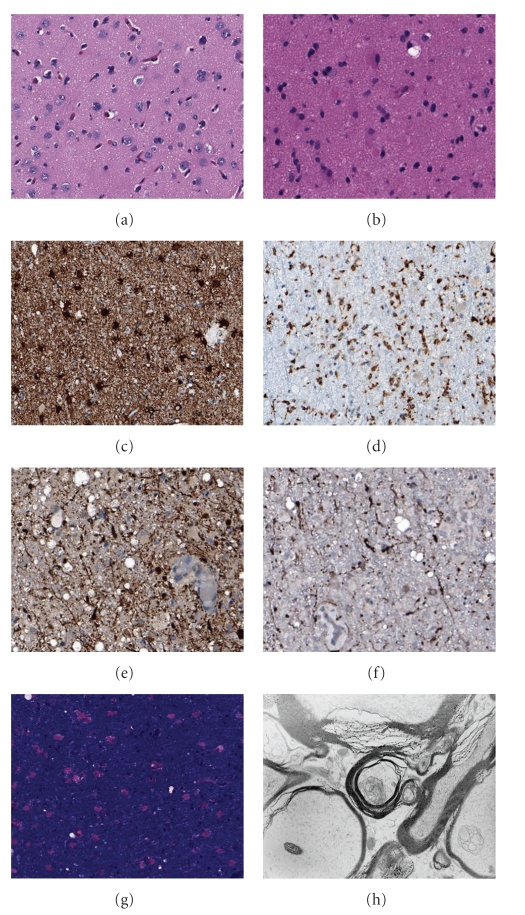
Cerebral cortex shows apparently normal neuronal density ((a) hematoxylin and eosin, ×200 original magnifications). The histological changes in the biopsy center in the white matter show increased cellularity with reactive gliosis ((b) hematoxylin and eosin, ×400; (c) anti-GFAP, ×200), microglial activation ((d) anti-CD68, ×200), and axonal changes, consistent with recent axonal injury ((e) anti-neurofilament, ×400; (f) anti-B-APP, ×200 all original magnifications) in the presence of abundant well-stained myelin ((g) luxol fast blue with hematoxylin and eosin, ×200). Ultrastructural evaluation confirms axonal degeneration with myelin preservation (h).

Initial brain biopsy was done 50 days from initial presentation to evaluate for hypoxic encephalopathy, toxic leukoencephlopathy, Creutzfeldt-Jakob disease, and metachromatic leukodystrophy. It was nondiagnostic as there was no white matter on the biopsy sample. A repeat biopsy of the right frontal lobe 12 days later revealed white matter changes including small vacuoles, axonal injury, diffuse reactive astrogliosis, and microglial proliferation. Luxol fast blue myelin stains revealed well-stained and preserved myelin. Immunohistochemical stains revealed immunoreactivity of axons in white matter area to b-APP and NF. White matter also showed strong immunoreactivity with CD 68 and GFAP (Figure 2).

A working diagnosis of DPHE was based on clinical history and neuroimaging findings. Fifty-six days from initial presentation, he was treated with intravenous methylprednisolone 500 mg twice daily for 5 days, amantadine 100 mg twice a day, vitamin C 500 mg twice a day, and vitamin E 800 mg three times a day. Following 5 days of therapy, the patient was more interactive and followed simple commands like open your month and touch examiner's finger. He recognized his wife and regained some long-term memories. He could again feed himself and used more words to interact and speak. After 1 week, methylphenidate was added to increase his participation in rehabilitation. Within 3 days he began having visual hallucinations consisting of spider webs and space ships around him. Both amantadine and methylphenidate were stopped and the visual hallucinations disappeared. He was able to perform his activities of daily living with verbal and procedural cueing. He was started on a slow taper of prednisone 80 mg, with incremental decreases by 20 mg every 2 weeks. He continued to show significant improvement during two weeks of inpatient rehabilitation therapy. At the time of discharge, he was independent in performing all activities of daily living and returned to his job as a computer engineer. A repeat MRI after 2 months was unchanged from his initial MRI (Figure 1). At 6-month followup following hospital discharge, neuropsychiatric testing revealed no deficits. He was doing well at 2-year followup clinic visit.

## 3. Discussion

DPHE is characterized by a delayed onset of neurological deterioration after hypoxic-ischemic brain injury [1]. The diagnosis of DPHE is based on typical clinical and radiological presentation, after excluding other conditions that may mimic DPHE [7]. MRI or CT brain studies in DPHE show diffuse periventricular white matter changes [8]. Classic neuropathological findings in DPHE associated with CO poisoning are characterized by diffuse symmetrical demyelination in white matter with preserved axons, U fibers, and perivascular white matter [9]. Although MRI brain in our patient showed diffuse white matter lesions, surprisingly histopathology showed axonal injury with intact myelin structure. Pathological findings in our patient are similar to those seen in toxic leukoencephlopathy including small vacuoles in the white matter, axonal injury, diffuse reactive astrogliosis, and microglial proliferation [10]. Difference in pathological changes in our patient indicates that methadone induced DPHE may be different in underlying pathophysiology from CO-induced DPHE and DPHE may represent a spectrum of disorders rather than a single clinical entity.

Even though impaired oligodendroglial function [11], reduced arylsulphatase A activity [12], altered immune response [13], and mitochondrial dysfunction [14] have been postulated in the etiology of DPHE, the underlying pathophysiology is unknown. In our patient, CSF MBP increased with time (2.8 NG/ML at initial presentation to 4.4 NG/ML at second admission) which may represent slow progressive axonal destruction. Delayed neurological symptoms after initial insult may be explained by this slow axonal destruction.

Methadone induced DPHE may be related to mitochondrial dysfunction as described in heroin-induced leukoencephlopathy. Mitochondrial dysfunction causing opioid related leukoencephlopathy has been suggested by electron microscopy, elevated lactate peak on magnetic resonance spectroscopy, and the clinical improvement following antioxidant therapy in few patients [14, 15]. 

There are no available controlled treatment trials for DPHE. Steroids have occasionally been used with some improvement in treatment of DPHE in children [2, 4]. In an animal study, dexamethasone was found to be effective in preventing histological brain damage and learning and memory impairment caused by hypoxic-ischemic insult [16]. 

Autoimmune response against MBP induced by aldehydes from lipid peroxidation [13] may play an important role in the pathophysiology of DPHE which may explain the success of methylprednisolone in our patient. Amantadine 100 mg twice a day may be tried if patient does not respond to intravenous steroids or if patient has prominent features of apathy and abulia [3]. High-dose vitamin C, vitamin E, and coenzyme Q10 may be tried for their possible antioxidant effects and role in mitochondrial disorders [12, 17]. Early and continued vigorous rehabilitation plays a very vital role in the management of these patients as seen in our patient [14]. The recovery and permanent neurological impairment from DPHE varies from series to series. Choi reported 75% full recovery in 1 year, and Shillito and Drinker reported only 50% full recovery within 2 years [1, 18]. Follow up MRI at 2 months was unchanged in our patient despite clinical improvement which goes along with a previous report of earlier clinical improvement and delayed improvement radiologically which may take up to 9 months [8]. As our patient was doing extremely well at 2-year follow up, we did not repeat his MRI brain although it would have been interesting to follow the progression of white matter lesions. 

The usage of methadone has increased significantly during the last decade with methadone-related deaths increasing 390% from 1999 to 2004 [19]. However, despite the widespread use of methadone and unintentional overdose on methadone, only 5 cases of methadone induced DPHE have been reported to date [2–6], suggesting that it is either uncommon or often goes unrecognized or unreported. Out of the 5 cases 4 were reported in children and only 1 was reported in a 24-year-old male. We expect that more DPHE cases will be seen with the widespread usage of methadone in pain clinics. Neurobehavioral changes may be the initial manifestation of DPHE. Clinicians, especially psychiatrists, ER clinicians, and neurologists, should be fully aware of this entity to avoid exposing the patient to extensive invasive diagnostic procedures. The diagnosis of DPHE can be made based on typical clinical presentation and neuroimaging findings without brain biopsy. Patients with DPHE should be considered for a trial of steroids with high doses of antioxidant in order to hasten clinical recovery.

## References

[1] Choi HS (1983). Delayed neurologic sequelae in carbon monoxide intoxication. *Archives of Neurology*.

[2] Anselmo M, Rainho AC, Vale MADOC (2006). Methadone intoxication in a child: toxic encephalopathy?. *Journal of Child Neurology*.

[3] Arciniegas DB, Frey KL, Anderson CA, Brousseau KM, Harris SN (2004). Amantadine for neurobehavioural deficits following delayed post-hypoxic encephalopathy. *Brain Injury*.

[4] Mills F, MacLennan SC, Devile CJ, Saunders DE (2008). Severe cerebellitis following methadone poisoning. *Pediatric Radiology*.

[5] Riascos R, Kumfa P, Rojas R, Cuellar H, Descartes F (2008). Fatal methadone intoxication in a child. *Emergency Radiology*.

[6] Zanin A, Masiero S, Severino MS, Calderone M, Da Dalt L, Laverda AM (2010). A delayed methadone encephalopathy: clinical and neuroradiological findings. *Journal of Child Neurology*.

[7] Filley CM, Kleinschmidt-DeMasters BK (2001). Toxic leukoencephalopathy. *New England Journal of Medicine*.

[8] Chen ZQ, Yang WJ, Cai L (2005). Clinical characteristics, CT and MRI findings for delayed encephalopathy after acute carbon monoxide poisoning. *Zhonghua Lao Dong Wei Sheng Zhi Ye Bing Za Zhi*.

[9] Grinker RR (1925). Über einen Fall von Leuchtgasvergiftung mit doppelseitiger Pallidumerweichung und schwerer Degeneration des tieferen Großhirnmarklagers. *Zeitschrift für die gesamte Neurologie und Psychiatrie*.

[10] Ryan A, Molloy FM, Farrell MA, Hutchinson M (2005). Fatal toxic leukoencephalopathy: clinical, radiological, and necropsy findings in two patients. *Journal of Neurology, Neurosurgery and Psychiatry*.

[11] Plum F, Posner JB, Hain RF (1962). Delayed neurological deterioration after anoxia. *Archives of Internal Medicine*.

[12] Weinberger LM, Schmidley JW, Schafer IA, Raghavan S (1994). Delayed postanoxic demyelination and arylsulfatase-A pseudodeficiency. *Neurology*.

[13] Thom SR, Bhopale VM, Fisher D, Zhang J, Gimotty P (2004). Delayed neuropathology after carbon monoxide poisoning is immune-mediated. *Proceedings of the National Academy of Sciences of the United States of America*.

[14] Kriegstein AR, Shungu DC, Millar WS (1999). Leukoencephalopathy and raised brain lactate from heroin vapor inhalation (“chasing the dragon”). *Neurology*.

[15] Wolters EC, Van Wijngaarden GK, Stam FC (1982). Leucoencephalopathy after inhaling “heroin” pyrolysate. *Lancet*.

[16] Ikeda T, Mishima K, Yoshikawa T (2002). Dexamethasone prevents long-lasting learning impairment following neonatal hypoxic-ischemic brain insult in rats. *Behavioural Brain Research*.

[17] Przyrembel H (1987). Therapy of mitochondrial disorders. *Journal of Inherited Metabolic Disease*.

[18] Shillito FH, Drinker CKa, Shaughnessy TJ (1936). The problem of nervous and mental sequelae in carbon monoxide poisoning. *Journal of American Medical Association*.

[19] National Drug Intelligence Center (2007). *Methadone Diversion, Abuse, and Misuse: Deaths Increasing at Alarming Rate*.

